# Retrospective Assessment of the Antigenic Similarity of Egg-Propagated and Cell Culture-Propagated Reference Influenza Viruses as Compared with Circulating Viruses across Influenza Seasons 2002–2003 to 2017–2018

**DOI:** 10.3390/ijerph17155423

**Published:** 2020-07-28

**Authors:** Sankarasubramanian Rajaram, Pirada Suphaphiphat, Josephine van Boxmeer, Mendel Haag, Brett Leav, Ike Iheanacho, Kristin Kistler, Raúl Ortiz de Lejarazu

**Affiliations:** 1 Seqirus UK Ltd., Maidenhead SL6 8AA, UK; pirada.suphaphiphat@seqirus.com (P.S.); jose.van_boxmeer@seqirus.com (J.v.B.); mendel.haag@seqirus.com (M.H.); baleav@gmail.com (B.L.); 2 Evidera Ltd., London W6 8BJ, UK; ike.iheanacho@evidera.com; 3 Evidera Inc., Waltham, MA 02451, USA; kristin.kistler@gmail.com; 4 School of Medicine, University of Valladolid, 47005 Valladolid, Spain; lejarazu@gmail.com

**Keywords:** influenza, vaccine, egg, cell, adaptation, mutation, effectiveness, antigen

## Abstract

Suboptimal vaccine effectiveness against seasonal influenza is a significant public health concern, partly explained by antigenic differences between vaccine viruses and viruses circulating in the environment. Haemagglutinin mutations within vaccine viruses acquired during serial passage in eggs have been identified as a source of antigenic variation between vaccine and circulating viruses. This study retrospectively compared the antigenic similarity of circulating influenza isolates with egg- and cell-propagated reference viruses to assess any observable trends over a 16-year period. Using annual and interim reports published by the Worldwide Influenza Centre, London, for the 2002–2003 to 2017–2018 influenza seasons, we assessed the proportions of circulating viruses which showed antigenic similarity to reference viruses by season. Egg-propagated reference viruses were well matched against circulating viruses for A/H1N1 and B/Yamagata. However, A/H3N2 and B/Victoria cell-propagated reference viruses appeared to be more antigenically similar to circulating A/H3N2 and B/Victoria viruses than egg-propagated reference viruses. These data support the possibility that A/H3N2 and B/Victoria viruses are relatively more prone to egg-adaptive mutation. Cell-propagated A/H3N2 and B/Victoria reference viruses were more antigenically similar to circulating A/H3N2 and B/Victoria viruses over a 16-year period than were egg-propagated reference viruses.

## 1. Introduction

Vaccination is recognized as being the most effective method of preventing seasonal influenza disease [1]. Over the past several decades, seasonal influenza vaccines have provided important public health benefits, in terms of reduced incidence of influenza-like illness (ILI), general practitioner visits, hospitalizations, and influenza-associated mortality [2,3,4]. Each year, in advance of the beginning of the influenza season, the World Health Organization (WHO) predicts which influenza viruses will be dominant in the upcoming season, and based on this assessment, recommends candidate vaccine viruses (CVVs) for inclusion in vaccines for the Northern and Southern Hemispheres [5].

Vaccine effectiveness (VE) data provide an estimate of the extent to which a vaccine protects against disease in a real-world setting, relative to no vaccination. Ultimately, the effectiveness of influenza vaccines depends on the interplay between the host, the vaccine, environment, and the circulating virus strains [6], with the effectiveness of the vaccine depending on the extent to which the virus strains it includes match those strains causing disease in the human population during a given season. Less than optimal VE can occur due to various factors, including mismatch between vaccine and circulating strains due to inaccurate strain predictions, as well as antigenic drift [7,8,9,10,11,12], original antigenic sin [13,14], and the viral propagation techniques used for vaccine manufacture (i.e., egg-based versus non-egg-based technologies). Particularly low VE has been observed in recent seasons against the A/H3N2 strain as compared with the VE observed for A/H1N1 and B strains. Influenza VE can vary considerably between strains and seasons, with strain-specific VE estimates of 33%, 23%, 54%, and 61% for antigenically similar A/H3N2, variant A/H3N2, B strains, and A/H1N1pdm09, respectively, observed over the past decade [15]. In the 2017–2018 season, circulating strains of A/H3N2 were antigenically distinct from egg-propagated reference viruses [16].

Antigenic drift has led to alterations in some A/H3N2 viruses, which inhibit them from replicating efficiently in embryonated chicken eggs [17,18], and this limits the number of viruses which can be used as CVVs. In addition to background antigenic drift, those viruses that can successfully be propagated in eggs can develop structural changes in the haemagglutinin (HA) receptor binding site (egg adaptation) during serial passage, further contributing to antigenic differences between circulating and reference vaccine strains [19,20,21,22,23]. Vaccine-induced antibody responses generated against egg-adapted HA can be less reactive against the HA expressed on circulating viruses [22,23], and this is thought to have played a role in the lower VE observed for A/H3N2 in recent seasons. Using influenza viruses propagated wholly in mammalian cell lines (or recombinant technology using non-egg substrates) avoids egg-adaptive mutations and can contribute to the better effectiveness or efficacy observed for cell culture-based (or recombinant [24]) vaccines [22,23,25,26].

Against this background, we performed a retrospective evaluation of antigenic similarity between circulating and WHO reference A/H1N1, A/H3N2, and B strain viruses propagated in either eggs or Madin–Darby canine kidney (MDCK) cells from the 2002–2003 to 2017–2018 influenza seasons. The main objectives of this study were as follows: to assess the degree of antigenic similarity between circulating viruses and egg-propagated reference viruses, and where possible, cell-propagated reference viruses; to identify and describe any trends or observations in the antigenic characterisation of the reference strains over time; and to facilitate future assessment of the relationship between levels of antigenic similarity and any observable differences in VE between different vaccines.

## 2. Materials and Methods

This retrospective analysis used publicly available reports from the Worldwide Influenza Centre (WIC; Francis Crick Institute, London, UK) for the 2002–2003 to 2017–2018 Northern Hemisphere and Southern Hemisphere influenza seasons. The WIC is one of six WHO collaborating centres worldwide, and these, together with 144 National Influenza Centres (NICs), form the WHO Global Influenza Surveillance and Response System. The collaborating centres monitor strain prevalence, perform antigenic and genetic characterizations of influenza viruses worldwide, and help inform the WHO annual recommendations on which CVVs to include in influenza vaccines. For this analysis, we used data from WIC interim and annual reports, which provided information on influenza viruses in Africa, the Eastern Mediterranean, Europe, and the Western Pacific (Hong Kong and China), based on virus samples received from sentinel and non-sentinel WHO influenza centre sites [27]. In this report, the term ”CVV” refers to influenza strains which were included in seasonal influenza vaccines (as recommended by the WHO); the term ”reference strains” refers to influenza strains against which circulating strains were tested by haemagglutination inhibition (HI) assay and plaque reduction neutralization assays (PRNA). Reference strains include CVVs, as well as other strains antigenically similar to the CVVs used as the reference strain in our comparison if the exact CVV strain was not included in the WIC report. All egg-propagated reference viruses selected as comparators were CVVs and antigenically like the vaccine viruses; whereas for cell-propagated reference viruses, antigenic likeness to recommended strains was undetermined for some seasons.

Antigenic similarity between reference viruses and circulating strains has principally been reported by the WIC as HI assay data comparing circulating viruses in terms of their reactivity to ferret sera raised against reference virus for a given season. Since February 2009, for the analysis of A/H3N2 viruses, these HI assays have routinely incorporated the neuraminidase (NA) inhibitor oseltamivir at a concentration of 20 nM, to avoid false-negative results due to the agglutination of viruses via NA protein. Where applicable, therefore, such use of oseltamivir is noted in the results of the present study. In recent seasons, A/H3N2 viruses have become increasingly difficult to assess by HI assay, due to their decreased ability to agglutinate red blood cells [28]. From 2008 onwards, PRNAs using ferret sera have also been used to complement the findings of the HI assays, and to estimate titres for isolates which could not be determined by HI assay.

The WIC reports present the antigenic characterisation data categorically, specifically, as the proportion of circulating viruses that show reactivity to the ferret antisera at titres relatively similar to, or different from, those at which reference viruses show reactivity (i.e., when the difference between these titres is ≤ two, four, or ≥eight dilutions, respectively). For the current analysis, antigenic similarity was defined as the circulating virus titre (at which reactivity was observed) being no more than four-fold lower than the reference virus titre. This definition is consistent with that used by the U.S. Centers for Disease Control and Prevention [29]. Viruses which did not meet the definition of antigenic similarity were considered to be antigenically different. The raw data listings within the WIC reports often included results from a small number of isolates tested outside of the periods or the geographic locations covered in the reports; and isolates grown in eggs were occasionally included as assay controls. Calculations using raw data (conducted when summarised reactivity data for ≤ two, four, or ≥ eight-fold differences in antisera titre were not presented within a WIC report) included only isolates exclusively grown in cells, and isolates harvested within the specific time period and geographic locations stated in the relevant reports. For A and B strain analyses, seasons were defined as Southern Hemisphere by time of year rather than by the geographical origin of circulating isolates (e.g., B/Victoria isolates were predominantly derived from Northern Hemisphere countries). All circulating viral isolates were passaged in MDCK/MDCK-SIAT1 cells.

We performed a qualitative comparison of the percentages of circulating virus isolates which were antigenically similar to the egg- and cell-propagated reference viruses for each influenza season. These percentages were calculated as a proportion of the total number of isolates tested by the WIC for each season. Because this was an exploratory analysis, no formal hypotheses were tested during the study.

## 3. Results

### 3.1. A/H3N2 Analysis

**Figure 1:**
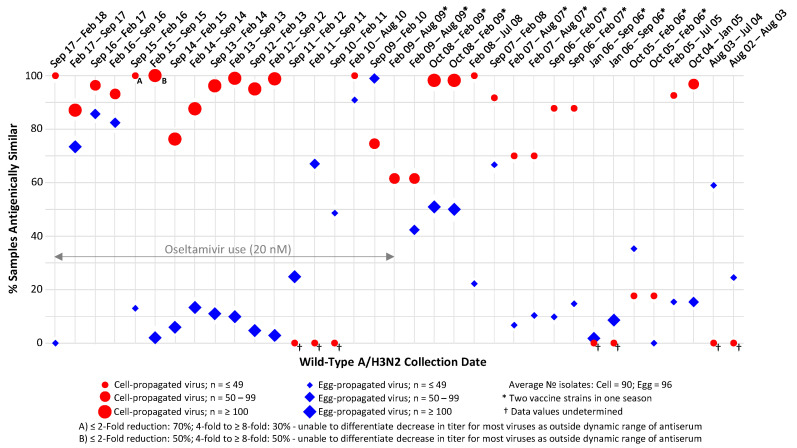
Proportions of circulating A/H3N2 isolates antigenically similar to egg- and Madin–Darby canine kidney (MDCK) cell-propagated reference viruses, as assessed by haemagglutination inhibition (HI) assay. Antigenic similarity is defined as circulating virus titre no more than 4-fold lower than reference virus titre. Data are displayed as percentage of the total number of isolates tested. Studied period August 2002 to February 2018.

HI assay data were available for cell-propagated reference viruses for 23 of 29 influenza seasons (Figure 1).

From the 2015 season onwards, less than half of the viruses could be assessed by HI assay due to loss of haemagglutination. In all but two of the seasons for which data were available for both egg- and cell-propagated reference viruses, a higher proportion of circulating A/H3N2 strains were antigenically similar to cell-propagated as compared with egg-propagated reference viruses (Figure 1). HI assay data demonstrated little or no antigenic similarity (i.e., present in <25% of isolates) between A/H3N2 isolates and egg-propagated reference viruses in 16 of the 29 (55%) seasons analysed. By comparison, cell-propagated reference viruses showed this low proportion of similarity (i.e., <25%) in only one of the 23 (4%) seasons for which data were available. Particularly large differences were observed in the period between the 2012 Southern Hemisphere and the 2015–2016 Northern Hemisphere seasons inclusive, for which 2–13% of isolates showed antigenic similarity to egg-propagated reference viruses, as opposed to 76–100% of cell-propagated reference viruses.

In two of the seasons analysed, a higher proportion of isolates showed antigenic similarity to egg-propagated as compared with cell-propagated reference viruses, i.e., the 2005–2006 Northern Hemisphere season (35% showed antigenic similarity to egg-propagated and 18% to cell-propagated CVVs [27]); and the 2009–2010 Northern Hemisphere season (99% showed antigenic similarity to egg-propagated as compared with 75% to cell-propagated CVVs [27]). In the 2009 Southern Hemisphere season, two antigenically similar reference strains were used, because both were listed as CVVs by the WHO. For this season, a higher percentage of isolates showed antigenic similarity to cell-propagated reference viruses against one of these strains, and 62% of isolates were antigenically similar to both egg- and cell-propagated reference viruses for the other strain. The difference between cell- and egg-propagated reference viruses in the three seasons between February 2016 and September 2017 was less than in other seasons. Further analysis of these seasons showed that differences between the egg- and cell-propagated group datasets were more pronounced if a stricter ≤ two-fold cut-off point for the difference in titres was employed (rather than ≤ four-fold), thus, more clearly demonstrating cell-propagated reference viruses to be more antigenically similar than egg-propagated reference viruses to circulating A/H3N2 viruses (Table 1).

Additionally, virus clade impacted these results, with greater differences observed between similarity to egg- and cell-propagated reference viruses for circulating 3C.2a clade viruses as compared with subdominant 3C.3a clade viruses.

**Figure 2:**
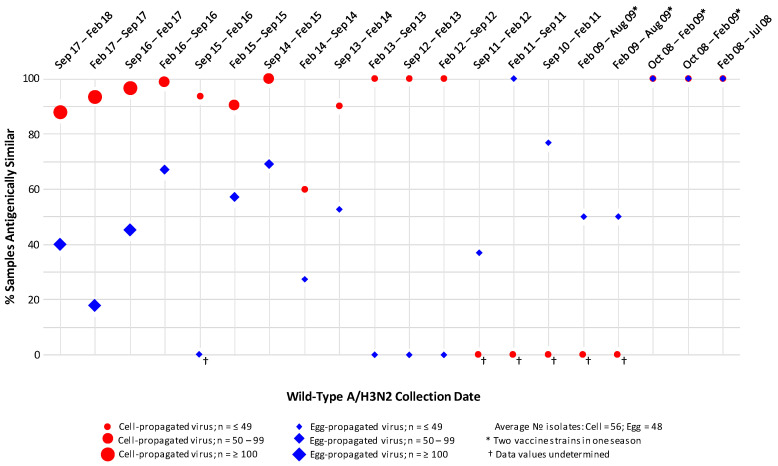
Proportions of circulating A/H3N2 isolates antigenically similar to egg- and MDCK cell-propagated reference viruses, as assessed by plaque reduction neutralisation (PRNA) assay. Antigenic similarity is defined as circulating virus titre no more than 4-fold lower than reference virus titre. Data are displayed as percentage of the total number of isolates tested. Studied period February 2008 to February 2018.

Assessment of antigenic similarity by PRNA demonstrated data trends similar to those observed by HI analysis (Figure 2); specifically, across seasons, 60–100% (median 98%) and 0–100% (median 50%) of cell- and egg-propagated viruses were antigenically similar to circulating isolates, respectively.

### 3.2. A/H1N1 Analysis

**Figure 3:**
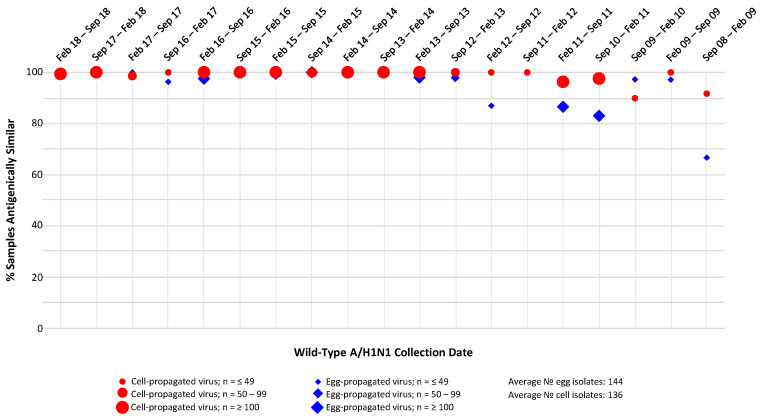
Proportions of circulating A/H1N1 isolates antigenically similar to egg- and MDCK cell-propagated reference viruses, as assessed by haemagglutination inhibition (HI) assay. Antigenic similarity is defined as circulating virus titre no more than 4-fold lower than reference virus titre. Data are displayed as percentage of the total number of isolates tested. Studied period September 2008 to September 2018.

HI assay analysis of the antigenic similarity between cell- and egg-propagated A/H1N1 reference viruses and the A/H1N1 viruses circulating in the environment during period September 2008–September 2018 (Figure 3), generally found a high degree of antigenic similarity for both egg- and cell-propagated A/H1N1 reference viruses, with little difference in levels of similarity observed between the two groups.

### 3.3. B/Victoria Strain Analysis

**Figure 4:**
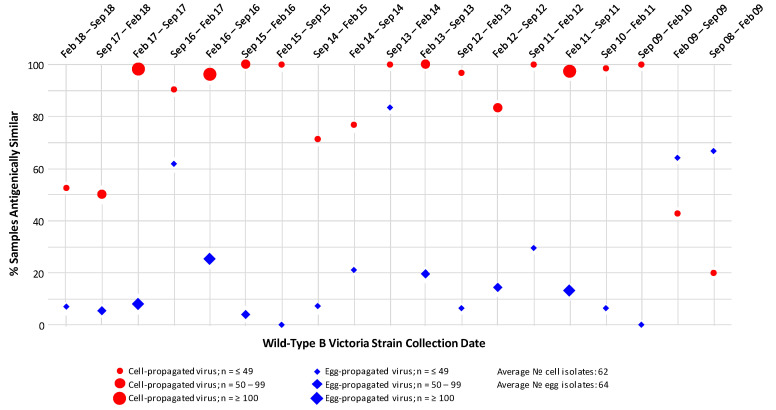
Proportions of circulating B/Victoria strain isolates antigenically similar to egg- and MDCK cell-propagated reference viruses, as assessed by haemagglutination inhibition (HI) assay. Antigenic similarity is defined as circulating virus titre no more than 4-fold lower than reference virus titre. Data are displayed as percentage of the total number of isolates tested. Studied period September 2008 to September 2018.

HI assay analysis of the antigenic similarity between cell- and egg-propagated B/Victoria strain reference viruses and the B/Victoria viruses circulating in the environment during the period September 2008–September 2018 (Figure 4), generally found a pronounced difference in the levels of antigenic similarity displayed by the egg- and cell-propagated reference viruses, with the higher levels of antigenic similarity observed for the cell-propagated viruses.

Over the 10 Northern Hemisphere influenza seasons (2008–2018), the proportions of cell-propagated reference viruses that were antigenically similar to circulating viruses ranged from 20% to 100% (with ≥90% being antigenically similar in seven of the 10 seasons). The proportions of egg-propagated reference viruses that were antigenically similar to circulating viruses ranged from 0% to 83% (with ≥ 90% being antigenically similar in none of the 10 seasons). With the exception of the 2008–2009 season, higher proportions of cell-propagated as compared with egg-propagated reference viruses were antigenically similar to circulating viruses. Data from the nine Southern Hemisphere influenza seasons (2009–2018) were comparable to the Northern Hemisphere data, with greater proportions of cell-propagated as compared with egg-propagated reference viruses being antigenically similar to circulating B/Victoria viruses in all seasons except one, namely February 2009–September 2009. Over the Southern Hemisphere influenza seasons, the proportions of cell-propagated reference viruses that were antigenically similar to circulating viruses ranged from 43% to 100% (and was ≥90% in five of the nine seasons). By comparison, the proportions of egg-propagated reference viruses that were antigenically similar to circulating viruses ranged from 0% to 64% (and was ≥90% in none of the nine seasons).

### 3.4. B/Yamagata Strain Analysis

**Figure 5:**
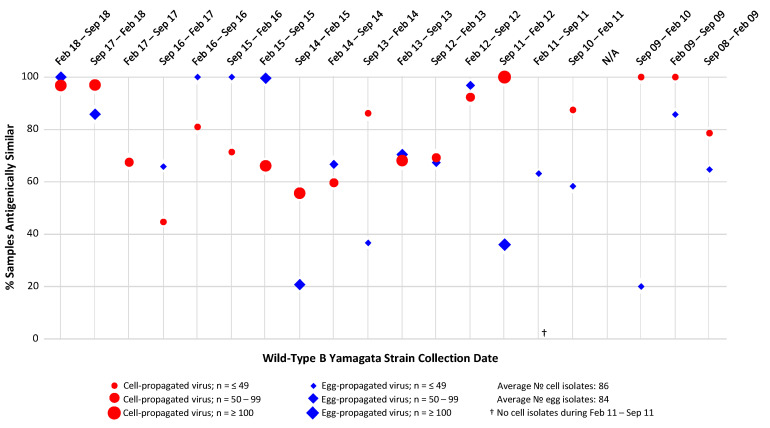
Proportions of circulating B/Yamagata strain isolates antigenically similar to egg- and MDCK cell-propagated reference viruses, as assessed by haemagglutination inhibition (HI) assay. Antigenic similarity is defined as circulating virus titre no more than 4-fold lower than reference virus titre. Data are displayed as percentage of the total number of isolates tested. Studied period September 2008 to September 2018.

HI assay analysis of the antigenic similarity between cell- and egg-propagated B/Yamagata strain reference viruses and the environmental B/Yamagata viruses present during the period September 2008–September 2018 (Figure 5), did not reveal any clear data trends distinguishing the egg- and cell-propagated viruses either from each other or the circulating viruses.

Across the 20 influenza seasons assessed (both Northern and Southern Hemisphere seasons), average percentages of similarity to circulating B/Yamagata isolates of 69% and 79% were observed for the egg- and cell-propagated viruses, respectively; therefore, overall levels of similarity to circulating B/Yamagata viruses were slightly higher for cell- than egg-propagated B/Yamagata reference viruses, a difference likely to be of no clinical significance. In eight (40%) of the 20 seasons assessed, egg-propagated reference viruses displayed higher similarity to circulating viruses than cell-propagated viruses. In 10 (50%) of the 20 seasons assessed, cell-propagated reference viruses displayed higher similarity to circulating viruses than did egg-propagated viruses.

## 4. Discussion

This exploratory, descriptive study is, to the best of our knowledge, the first such analysis to assess the antigenic similarity between circulating strains of influenza, and egg- and cell-propagated reference viruses longitudinally over several Northern and Southern Hemisphere influenza seasons, both before and after the 2009 pandemic outbreak. The data presented in this report show that between 2002 and 2018, cell-propagated A/H3N2 and B/Victoria strain reference viruses were more often antigenically similar to A/H3N2 and B/Victoria viruses circulating in the environment than were egg-propagated viruses. For A/H1N1 and B/Yamagata reference viruses, similar proportions of samples showed antigenic similarity in both egg- and cell-propagated viruses. This distinction between A/H3N2 and B/Victoria versus A/H1N1 and B/Yamagata data is consistent with the possibility that there were higher rates or degrees of HA egg adaptation in A/H3N2 and B/Victoria strains than in A/H1N1 and B/Yamagata strains. It is also possible that, unlike A/H1N1 and B/Yamagata, A/H3N2 and B/Victoria are more susceptible to egg adaptations at specific regions of the HA molecule that significantly affect antigenicity (i.e., major epitopes). Analyses have shown some degree of similarity in phylodynamics to exist between A/H3N2 and B/Victoria viruses, as well as between A/H1N1 and B/Yamagata viruses [30]. A/H3N2 and B/Victoria exhibit limited genetic diversity at a given time point as compared with A/H1N1 and B/Yamagata viruses, which is indicative of frequent selective bottlenecks due to the serial replacement of circulating strains, as expected under continuous antigenic drift; this evolutionary pattern is consistent with the fact that A/H3N2 and B/Victoria are more susceptible to adaptive mutations on encountering the selective pressures of surviving in eggs [30].

The decrease in A/H3N2-specific VE observed over recent seasons is particularly noteworthy, because A/H3N2 is responsible for more severe cases of disease and higher rates of mortality than are other strains, with the highest burden of A/H3N2 disease occurring in adults aged 65 years or older [31,32]. Improving the effectiveness of influenza vaccines against circulating A/H3N2 strains is, therefore, a public health priority. The A/H3N2 data generated in this study frequently demonstrated considerable variance in the percentages of antigenic similarity observed within seasons, particularly between 2012 and 2016. In more than half of the seasons analysed, there was little (<25%) or no antigenic similarity between egg-propagated reference and circulating A/H3N2 viruses when analysed by HI assay. In seasons 2012–2016, approximately 100% of cell- and 20% of egg-propagated A/H3N2 reference viruses were shown to be antigenically similar to circulating viruses. In the 2016–2017 season, an egg-adaptive mutation (T160K) which arose during propagation of the A/H3N2 strain resulted in deglycosylation of the HA antigenic B site and altered antibody to HA glycoprotein binding; this mutation could have contributed to the low A/H3N2-specific VE of 34% observed during this season, despite a 95% match between circulating A/H3N2 isolates and the CVV [23,27,33,34,35,36]. The CVV used for the 2016–2017 cell-derived vaccines was passaged in eggs, which led to egg-adaptive mutations persisting and being present in the seed virus, and therefore also in the cell-based vaccine [36]. The A/H3N2-dominant 2017 season in Australia saw A/H3N2 isolates which were more likely to be ”low-reacting” to egg- than to cell-propagated reference viruses [37], suggesting that recombinant or cell-based vaccines could confer advantages over egg-derived vaccines in A/H3N2 dominant seasons with egg adaptation. In the 2017–2018 Northern Hemisphere season, an inactivated, cell culture-derived, quadrivalent vaccine, Flucelvax^®^ (Seqirus USA Inc., Summit, NJ, USA) was manufactured for the first time using an exclusively cell-propagated A/H3N2 seed virus [38]. During this influenza season, A/H3N2 was the predominant circulating strain in the USA, and the overwhelming majority (93%) of these strains were antigenically similar to the cell-propagated reference virus used in the vaccine [16]. Importantly, the cell-derived vaccine was shown to have a relative VE of 10.7% as compared with an egg-derived quadrivalent vaccine in individuals aged 65 years or above for season 2017–2018 [26]. In the same season, Bruxvoort et al. estimated the absolute VE of cell- and egg-derived vaccines (against laboratory-confirmed hospitalization for any influenza) in a population <65 years of age to be 36% and −11%, respectively [39]. This difference was even more pronounced for A/H3N2, although confidence intervals were wide. For the same season, Klein et al. estimated absolute VE against influenza B to be 41% for cell- and 10% for egg-derived vaccines in a population 4–64 years of age [40]. Another study which compared the protective efficacy of a recombinant vaccine with that of a standard-dose, egg-derived, quadrivalent vaccine in adults (*N* = 9003), was performed during the A/H3N2-dominant 2014–2015 season (during which the effectiveness of many vaccines was particularly low due to antigenic mismatch between circulating and vaccine viruses) [24]. The probability of ILI in this study was 30% lower in recipients of recombinant vaccine as compared with egg-derived vaccine (95% CI 10–47, *p* = 0.006).

It is notable that the proportion of circulating A/H3N2 isolates showing antigenic similarity to A/H3N2 egg- and cell-propagated reference viruses was very similar for the three seasons from February 2016 to September 2017 (HI data); however, when analysed with a stricter definition of antigenic similarity (≤two-fold lower titre, Table 2) or by PRNA, differences between the egg- and cell-propagated datasets became apparent.

One factor which could have affected the assessments of antigenic similarity in these three seasons was the pool of virus strains that could be characterised by HI assay. For example, in the first season (2016 Southern Hemisphere), ~80% of isolates were clade 3C.2a (similar to the vaccine strain), while the other ~20% were 3C.3a. However, two-thirds of the 3C.2a viruses did not agglutinate red blood cells. Antisera raised against the cell-propagated reference virus recognised both 3C.2a and 3C.3a equally well, but antisera raised against the egg-propagated reference strain recognised the 3C.3a viruses better than the 3C.2a viruses (whereas 3C.2a was the dominant population) [27]. Therefore, the reported percentages from the HI assay based on the strains tested may not be truly representative of how well vaccines containing the egg-propagated reference viruses would cover circulating isolates and could be artificially high. Additionally, in two of the other seasons analysed (2005–2006 Northern Hemisphere and 2009–2010 Northern Hemisphere), a higher proportion of isolates showed antigenic similarity to egg- than with cell-propagated reference viruses. One possible explanation for this is that the same egg- and cell-propagated reference viruses were not available for these two seasons, and therefore data were selected for the genetically closest strain based on the phylogenetic tree analysis of the HA gene (also included in the WIC reports). These differences may have meant that the cell-propagated reference strain was less representative, leading to the anomalous findings for these two seasons.

This study had three main limitations. The first of these was an inability to assess all viruses accurately by HI assay, particularly in more recent seasons. Structural changes in many A/H3N2 viruses resulted in a decreased ability to agglutinate red blood cells, which made these viruses difficult to characterise by HI assay [41,42]; in addition, a NA mutation led to NA-mediated agglutination, which could have confounded the HI data (by somewhat masking the decreased haemagglutination inhibition) before oseltamivir was routinely used at the WIC from 2009 onwards [42,43]. The second limitation was that the overall number of isolates included in the analysis represented only a small proportion of all those received by the WHO collaborating centres over the influenza seasons analysed. In some cases, data on the egg- and cell culture-propagated version of the same reference virus strain were not available in the reports to be used for direct comparison. It should also be noted that the PRNA data were limited by small sample sizes for seasons prior to 2014. In addition, the PRNA data were limited by WIC processes for selecting isolates for testing, i.e., rather than being randomly selected, isolates were chosen specifically to include A/H3N2 clades circulating during a given season, but not necessarily in proportion to the dominance of each clade within the population (Dr John McCauley [WIC], personal communication, 2019). The third study limitation was the use of ferret rather than human sera in the PRNA and HI assays. Ferret sera is used as standard by all WHO collaborating centres for both assays; however, evidence suggests that the antigenic reactivity of viruses can vary depending on the origin of the sera used [44,45]. The potential impact of using ferret rather than human sera in the PRNA and HI assays should be taken into consideration when interpreting the results of any future research.

Some of the strengths of this study include the longitudinal nature of the analyses, which allows for the assessment of trends over multiple seasons, rather than individual seasons in isolation. The assessment of viruses by both PRNA and HI assays (rather than HI assay alone) is also a study strength, particularly for the more recent seasons where most viruses could not be assessed by HI assay. Further to previous studies and the existing literature [46], future research is warranted to evaluate the potential links between antigenic similarity, levels of VE, and burden of disease. A longitudinal dataset such as that presented in this report could be useful in the study of the relationships among the levels of antigenic similarity and VE in terms of influenza-associated hospitalizations or ILI incidence. Preferably, this would be assessed in a specific region or country such as the USA, which has a large population and high-quality surveillance procedures.

## 5. Conclusions

In summary, the results of this study demonstrate higher levels of antigenic similarity between cell-propagated A/H3N2 and B/Victoria reference viruses and circulating viruses, than between egg-propagated A/H3N2 and B/Victoria reference and circulating viruses in both Northern and Southern Hemispheres over a 16-year period; no similar trend was observed for A/H1N1 and B/Yamagata viruses. Further research is required in this area to better understand the impact of the more contemporary, non-egg-based technologies on influenza vaccine effectiveness.

## Figures and Tables

**Table 1:** Percentages of circulating A/H3N2 viruses which showed ≤ 2-fold and 4-fold differences (HI assay) to cell- and egg-propagated reference virus titres for the February 2016–February 2017 influenza seasons.

Influenza Season	Reference Virus	≤2-Fold	4-Fold
Feburary 2016–September 2016	Cell-based	69%	24%
Feburary 2016–September 2016	Egg-based	35%	47%
September 2016–Feburary 2017	Cell-based	52%	45%
September 2016–Feburary 2017	Egg-based	38%	48%

**Table 2:** Percentages of circulating 3C.2a and 3C.3a A/H3N2 viruses which showed ≤ 2-fold and 4-fold differences (HI assay) to cell- and egg-propagated reference virus titres for the February 2016–September 2016 influenza seasons.

Clade	Reference Virus	≤2-Fold	4-Fold
3C.2a	Cell-based	60%	31%
3C.2a	Egg-based	19%	50%
3C.3a	Cell-based	76%	20%
3C.3a	Egg-based	64%	36%
